# Supplementary material for: Nationwide Real-World Data of Microsatellite Instability and/or Mismatch Repair Deficiency in Cancer: Prevalence and Testing Patterns
Source: Diagnostics (Basel). 2024 May 22;14(11):1076. doi: 10.3390/diagnostics14111076 (PMC11171982; doi:10.3390/diagnostics14111076)
Supplement: Supplementary file 1 [file diagnostics-14-01076-s001.zip › diagnostics-2986900-supplementary.pdf]

MSI testing methodology

- Hematoxylin and eosin-stained sections of formalin-fixed and paraffin-embedded (FFPE) tumor biopsies from all samples were microscopically reviewed and the tumor area was marked by a pathologist. The QIAsymphony DNA Midi Kit (Qiagen) was utilized to isolate genomic DNA from both tumor and healthy tissue. Five polymorphic markers (AT-25, BAT-26, D5S346, D17S250, and D2S123) were analyzed according to the Amsterdam meeting criteria (PMID: 9823339). In summary, the microsatellite sites were amplified using a 25μl multiplex PCR (QIAGEN Multiplex PCR Kit, Qiagen) in conjunction with 100ng of DNA and five pairs of fluorescently labeled primers as previously described (PMID: 9354436). The amplified fragments were separated by size using capillary electrophoresis on an Applied Biosystems 3130 genetic analyzer (Thermo Fischer Scientific). The lengths of the microsatellite sites were compared between tumor and normal tissue using the GeneMapper software v.4.0 (Thermo Fischer Scientific). A tissue was considered MSI-H (microsatellite-high) when instability was observed in two or more polymorphic markers of the microsatellites analyzed. MSI-L (microsatellite-low) tumors exhibited instability in one of the examined microsatellites, while microsatellite-stable (MSS) tumors did not exhibit instability in any of the microsatellites under investigation.

  For each microsatellite sequence (BAT25, BAT26, NR21, NR24, NR27), DNA from cancer tissue was examined with PCR HRM (high resolution melting analysis), based on the literature (DOI: 10.1373/clinchem.2010.150680) using the Qiagen Type-it HRM PCR Kit on a Cobas z 480 machine. The division of results into MS-Stable and MSI-H, according to this methodology, was performed as follows:

  1. MSI – HIGH ≥ 2 microsatellite sequences
  2. MS – STABLE < 2 microsatellite sequences

- MSI testing carried out by PCR with Fragment Analysis was performed as previously described (doi: 10.3390/cancers15020353). Fluorescent labeled DNA fragments from each tumor and the normal tissues, separated by capillary electrophoresis, were analyzed. Five mononucleotide markers (NR-21, BAT-26, BAT-25, NR-24, and MONO-

27) were used in comparison with an internal standard using the Promega MSI Analysis System. The MSI status was defined in accordance with the Bethesda guidelines. Detection of the amplified loci used the ABI Prism 3100 genetic Analyzer with Data Collection Software, Version 4.1. Instability was defined as any change in length of a detection loci of more than two base pairs when compared to the same marker in the normal sample. MSI-H was defined when two or more of the five loci were unstable, MSI-Low when one marker was unstable, and MSI-Stable (MSS) when no unstable loci were detected.

- Microsatellite instability was estimated according to the National Cancer Institute recommendations (PMID: 9823339). Briefly, for each patient, DNA was isolated from peripheral blood (QIAamp DNA Mini kit, QIAGEN) and FFPE tissue (AmoyDx® FFPE DNA Kit for FFPE tissue, Amoy Diagnostics Co., Ltd. or QIAamp DNA Mini kit, QIAGEN following deparaffinization with standard procedures) according to the manufacturer. PCR was performed to amplify D2S123, D17S250, D5S346, Bat-25, Bat-26 in both DNA samples. PCR products were separated by fragment analysis (CEQ 8000 Genetic Analysis System, Beckman Coulter) and fragment profiles from peripheral blood (reference tissue) and FFPE tissue were compared to determine MSI status.

- The tissue block was examined by a specialized pathologist and it was verified that the chosen section was the most suitable for the conduction of molecular testing (percentage of cancer cells, necrosis, normal tissue). For further enrichment of the sample in cancer cells, microdissection was implemented, by subtracting all other necrotic cells or normal tissue, that could affect the validity of the results. From the paraffin-embedded tissue, genomic DNA was isolated with the RecoverAll™ Total Nucleic Acid Isolation Kit. The molecular test was conducted with the real-time PCR and hybridization methods in the Agilent AriaDx system. The test was conducted with the certified EasyPGX® ready MSI Kit (CE/IVD) (Diatech), which is designed to detect mutations in eight microsatellite genetic loci (BAT25, BAT26, NR21, NR22, NR24, NR27, CAT25 and MONO27), according to updated Bethesda criteria and latest evidence. For every microsatellite analyzed, DNA from the tumor tissue was compared

with normal DNA. The results were extracted as per the following scheme: MS — STABLE = 0 unstable microsatellites and MSI-H) ≥ 2 unstable microsatellites.

➢ The Ion AmpliSeq Microsatellite Instability Panel (Thermo Fischer Scientific) was utilized to perform NGS-based microsatellite instability analysis on tumor-only and tumor-normal samples, as previously described (PMID: 33853586). This panel analyzed 76 markers to determine the MSI status. The Ion Gene Studio S5 Prime System Next Generation Sequencing platform (Thermo Fisher Scientific) was utilized for the sequencing procedure. The sequencing data were analyzed by Torrent Suite™ software (Thermo Fischer Scientific) using the "MSICall" plugin. The assessment yielded outcomes for every individual microsatellite and produced an MSI score. Positive MSI status was assigned to a sample when the MSI score was greater than 30.

➢ DNA was extracted from tissue sections reviewed by a pathologist. A DNA library targeting BAT25, BAT26, NR21, NR22, and NR27 quasimonomorphic markers was generated using an AmpliSeqTM Panel, sequenced by semiconductor-based next generation sequencing technology on an Ion Torrent PGM and analyzed with Ion Suite (v5.12.3) and MiSuite (v1.2) software. Each marker was called as stable (one allele detected) or unstable (more than one allele detected). Depending on the total number of unstable markers, the result was expressed as microsatellite stable (MSS, no unstable marker), microsatellite unstable low (MSI-LOW, one marker unstable) or microsatellite unstable high (MSI-H, more than one marker unstable). For each marker, target coverage was 1000 reads and minimum coverage for reporting was 100 reads. The minimum detectable mutant/unstable allele ratio was approximately 10%. Testing was conducted on tumor tissue (neoplastic cell content >20%) and a matched normal control (normal surrounding tissue or alternative), if available.

➢ With the Biocartis Idylla™ System platform and the Idylla™ MSI Test (CE-IVD), detection of seven monomorphic biomarkers, including ACVR2A, BTBD7, DIDO1, MRE11, RYR3, SEC31A, and SULF2, was performed. For this specific technique, PCR amplification for each specific biomarker resulted in a characteristic fluorescence profile.

The integrated software processed all fluorescence profiles in the test and compared the result for the normal and mutated sample. Thus, the MSI score for a given valid biomarker profile was calculated. A score of < 0.5 (score cut-off value 0.5) was considered "Mutation not detected", while the presence of mutated PCR products in the sample resulted in a fluorescence profile with an MSI score ≥ 0.5 to 1, indicated as "Mutation detected". The displayed MSI score was intended for informational purposes only.

Immunohistochemistry (IHC) for Mismatch Repair (MMR) Proteins

- IHC was carried out on 2 μm thick paraffin sections of tumors using a Bond Max autostainer (Leica Microsystems). Antibodies and conditions were as follows: MLH1, clone ES05 (Monosan, Uden, Netherlands) at 1:80 dilution; MSH2, clone 25D12 (Novocastra/Leica Microsystems, Wetzlar, Germany) at 1:40 dilution; MSH6, clone EP49 (DAKO, Glostrup, Denmark) at 1:60 dilution; and PMS2, clone M0R4G (Novocastra/Leica Microsystems) at 1:50 dilution. Diaminobenzidine was used as chromogen for protein–antibody complex visualization. Stains were evaluated by pathologists for all tumor and normal tissue sections, along with external controls for assessing method performance. Each tumor was evaluated for nuclear staining intensity and distribution of positive cells at 200X and 400X magnification. Cases were considered as pMMR (proficient MMR) if any degree of nuclear expression was observed for all four proteins in the neoplastic cells and dMMR if no IHC nuclear expression was seen for any of the four proteins.

- The evaluation of the immunohistochemical expression of the four MMR proteins, namely MLH1, MSH2, MSH6, and PMS2, followed CAP guidelines. The non-neoplastic elements were first examined to confirm positive staining and ascertain that the internal control for each case had worked appropriately. Any staining in the tumor nuclei was considered preserved normal protein expression. Pathologic loss of protein expression was recorded if no staining was seen in the neoplastic nuclei, provided that the

interspersed non-neoplastic cells (endothelial cells, inflammatory cells, and stromal elements) were expressing the protein.

- From each representative archival paraffin embedded tissue block, four 4 μm tissue sections were cut and assayed for MLH1, MLH2, MSH6, PMS2, by IHC. The following antibodies were used: FLEX Ready-To-Use rabbit and mouse monoclonal antibodies against the mismatch repair proteins (clones ES05, FE11, EP49, and EP51 for MLH1, MLH2, MSH6, and PMS2, respectively, DAKO). For each antibody, the appropriate positive and negative controls were used. Tumors were considered dMMR if at least one of the four MMR proteins showed complete absence of immunoreaction, whereas the remaining cases were considered pMMR.

- The immunohistochemical examination of MMR proteins was performed by the Ventana Benchmark XT automated stainer (Roche Diagnostics, IVD) according to the manufacturer. The antibody clones used were M1 against MLH1, G219-1129 against MSH2, SP 93 against MSH6, and A16-4 against PMS2 proteins (VENTANA MMR RxDx Panel, Roche Diagnostics).

- A four-antibody panel of MMR proteins including MLH1, MSH2, MSH6, and PMS2 was performed by using the DAKO EnVision method on the representative paraffin-fixed tissue blocks. Three micrometer-thick tissue sections were deparaffinized in xylene, rehydrated in alcohol, and washed in distilled water. The formalin-fixed paraffin-embedded tissue sections were pretreated with heat-induced epitope retrieval (HIER) at 97 °C for 35–40 min at high pH (50×). The slides were then incubated with the following antibodies: MLH1, MSH2, MSH6, and PMS2. All the antibodies were ready-to-use monoclonal antibodies (MSH6 clone, EP49; PMS2 clone, EP51; MSH2 clone, FE11; MLH1 clone, ES05). Slides were placed on a Dako Autostainer Link instrument, where the immunohistochemical staining procedure was performed using the Dako EnVision TM FLEX detection system. The slides were removed from the autostainer and washed in running tap water. Then, the slides were counterstained with EnVision TM FLEX hematoxylin and blued in running tap water. Finally, the slides were

dehydrated through graded series of ethanol solutions (70 o , 80 o , 96 o , 100 o , 100 o), cleared in xylene, and mounted with DPX mounting medium. According to the CAP protocol for IHC interpretation, any nuclear staining even patchy is taken as “no loss of expression” and only absolute absence of nuclear staining should be considered “loss of expression” provided internal controls are positive. Adjacent normal epithelium, lymphocytes, and stromal cells served as positive internal controls.

- IHC was performed in a Ventana XT instrument using MLH1, MSH2, and MSH6 antibodies from DAKO, Ready to Use; while, for PMS2, the corresponding antibody from Ventana used was clone A16-4 (protocol comprised the Opti-View amplification kit from Ventana).

- Immunohistochemically, a qualitative determination of the expression of the MLH1, PMS2, MSH2, and MSH6 proteins was performed using monoclonal antibodies specific for the binding and staining for the above proteins. Staining was visible under the optical microscope. The available test (CE-IVD, FDA approved) was the VENTANA MMR RxDx Panel, applied on sections derived from formalin-fixed and paraffin-embedded (FFPE) tissue and was performed using the VENTANA BenchMark ULTRA platform. Each VENTANA anti-MLH1 (M1), VENTANA anti-PMS2 (A16-4), VENTANA anti-MSH2 (G219-1129), and VENTANA anti-MSH6 (SP93) antibody was a monoclonal antibody produced against a full-length recombinant protein. Each antibody bound to each MMR protein in FFPE tissue sections. Secondary antibody binding followed, and the complex was visualized using horseradish peroxidase (HRP) enzyme binding. This final antibody–enzyme complex was visualized and visible under the optical microscope. Each step of the assay followed the manufacturer's protocol as listed for each antibody (Ventana Roche, 1015758US Rev H, 1015761US Rev H, 1015760US Rev H, 1015759US Rev H, 2022-06-21).

- Expression of MMR proteins MLH1, PMS2, MSH2, and MSH6 was evaluated by IHC. Intensity and percentage were recorded for each protein. MMR protein expression was considered positive if ≥1% positive nuclei with mild to strong intensity were counted;

negative if internal controls (stromal cells and lymphocytic infiltrates) were positive and tumor cells were completely negative or exhibited any staining <1%; noninformative if tumor cells were negative and internal controls were negative. Tumors were classified as pMMR if informative and positive for all four MMR proteins. Tumors were classified as dMMR in the absence of expression of at least one of the four proteins.
